# Establishment and experimental validation of an immune miRNA signature for assessing prognosis and immune landscape of patients with colorectal cancer

**DOI:** 10.1111/jcmm.16696

**Published:** 2021-06-07

**Authors:** Zaoqu Liu, Taoyuan Lu, Yanli Wang, Dechao Jiao, Zhaonan Li, Libo Wang, Long Liu, Chunguang Guo, Yanan Zhao, Xinwei Han

**Affiliations:** ^1^ Department of Interventional Radiology The First Affiliated Hospital of Zhengzhou University Zhengzhou China; ^2^ Department of Cerebrovascular Disease Zhengzhou University People's Hospital Zhengzhou China; ^3^ Department of Hepatobiliary and Pancreatic Surgery The First Affiliated Hospital of Zhengzhou University Zhengzhou China; ^4^ Department of Endovascular Surgery The First Affiliated Hospital of Zhengzhou University Zhengzhou China

**Keywords:** Colorectal cancer, Immunotherapy, MicroRNA, Prognosis

## Abstract

As essential regulators of gene expression, miRNAs are engaged in the initiation and progression of colorectal cancer (CRC), including antitumour immune response. In this study, we proposed an integrated algorithm, ImmuMiRNA, for identifying miRNA modulators of immune‐associated pathways. Based on these immune‐associated miRNAs, we applied the LASSO algorithm to develop a reliable and individualized signature for evaluating overall survival (OS) and inflammatory landscape of CRC patients. An external public data set and qRT‐PCR data from 40 samples were further utilized to validate this signature. As a result, an immune‐associated miRNA prognostic signature (IAMIPS) consisting of three miRNAs (miR‐194‐3P, miR‐216a‐5p and miR‐3677‐3p) was established and validated. Patients in the high‐risk group possessed worse OS. After stratification for clinical factors, the signature remained a powerful independent predictor for OS. IAMIPS displayed much better accuracy than the traditional clinical stage in assessing the prognosis of CRC. Further analysis revealed that patients in the high‐risk group were characterized by inflammatory response, abundance immune cell infiltration, and higher immune checkpoint profiles and tumour mutation burden (TMB). In conclusion, the IAMIPS is highly predictive of OS in patients with CRC, which may serve as a powerful prognostic tool to further optimize immunotherapies for cancer.

## 1 INTRODUCTION

Colorectal cancer (CRC) is one of the most prevalent gastrointestinal malignancies with adverse prognosis. Currently, it has become the fourth most deadly tumour globally and accounts for approximately 10% of annual cancer‐associated deaths.^1^ Surgery or endoscopic treatment is the cornerstone of curative therapy for patients with CRC. Chemoradiotherapy such as fluoropyrimidine combined with radiotherapy can benefit inoperable patients in clinical practice.^2^ Though recent advances in various treatments, the overall survival (OS) of CRC remains unsatisfactory.^3^ More complicated, owing to the high heterogeneity of CRC, it is also challenging to predict prognosis and make clinical decision individually.

Over the past decade, immunotherapy has illustrated tremendous sensation owing to its remarkable efficacy in the treatment of solid tumours, such as melanoma, non‐small‐cell lung cancer, gastric carcinoma, head and neck squamous cell carcinoma, renal cell carcinoma and CRC.^4^, ^5^ Immune checkpoint inhibitors (ICIs) aim to help the immune system recognize and attack cancer cells by acting on the primary targets including programmed death‐ligand 1 (*PD‐L1*), programmed death 1 (*PD‐1*) and cytotoxic T‐lymphocyte‐associated protein 4 (*CTLA‐4*).^6^ In CRC, ICI therapy was approved in 2017 for the treatment of patients with DNA mismatch repair deficient (dMMR) or advanced microsatellite instability (MSI). However, only a subset of patients with CRC could benefit from immunotherapy.^7^ Therefore, it is imperative to pursue reliable biomarkers that are competent to accurately assess immunotherapy response.

MiRNAs are a series of small non‐coding single‐stranded RNA molecules that can post‐transcriptionally regulate gene expression by binding to target mRNAs and inhibiting the translation. MiRNAs have profound impacts on the prognosis of CRC. For instance, overexpressed *miR‐21* and *miR‐200c* have been demonstrated to be indicators of adverse prognosis, and high *miR‐150* level was significantly associated with better prognosis in CRC.^8^ Moreover, miRNAs play non‐negligible roles in immune infiltration and immunotherapy. Previous studies have reported that the down‐regulation of *miR‐506‐3p* led to macrophage recruitment in CRC and the overexpression of *miR‐200c* promoted the expansion and immune suppressive activity of myeloid‐derived suppressor cells (MDSCs).^9^, ^10^ Several miRNAs such as *miR‐21*, *miR‐20b* and *miR‐130b* are dramatically up‐regulated in advanced CRC and inhibit *PTEN* expression, further resulting in *PD‐L1* overexpression, which suggests that miRNA‐PD‐L1 axis might be a therapeutic target for CRC.^11^ Therefore, miRNAs have great implications for the prognosis and immunotherapy.

Considering the significant implications of miRNAs on the prognosis and immunotherapy of CRC, we proposed an integrated algorithm, ImmuMiRNA, for identifying miRNA modulators of immune‐associated pathways. Based on these identified immune‐associated miRNAs, the LASSO algorithm was applied to establish a risk signature for evaluating OS of CRC patients. As a result, an immune‐associated miRNA prognostic signature (IAMIPS) consisting of three miRNAs was established and further validated in an external public data set and qRT‐PCR data from 40 samples. We also investigated the immune landscape, immune checkpoint profiles and tumour mutation burden (TMB) of the signature. Initial construction of an IAMIPS for patients with CRC will facilitate the complex underlying mechanisms between immune‐associated miRNAs and prognosis of CRC and may advance optimize immunotherapies for patients with CRC.

## 2 MATERIALS AND METHODS

### 2.1 Public data set collection

**FIGURE 1:**
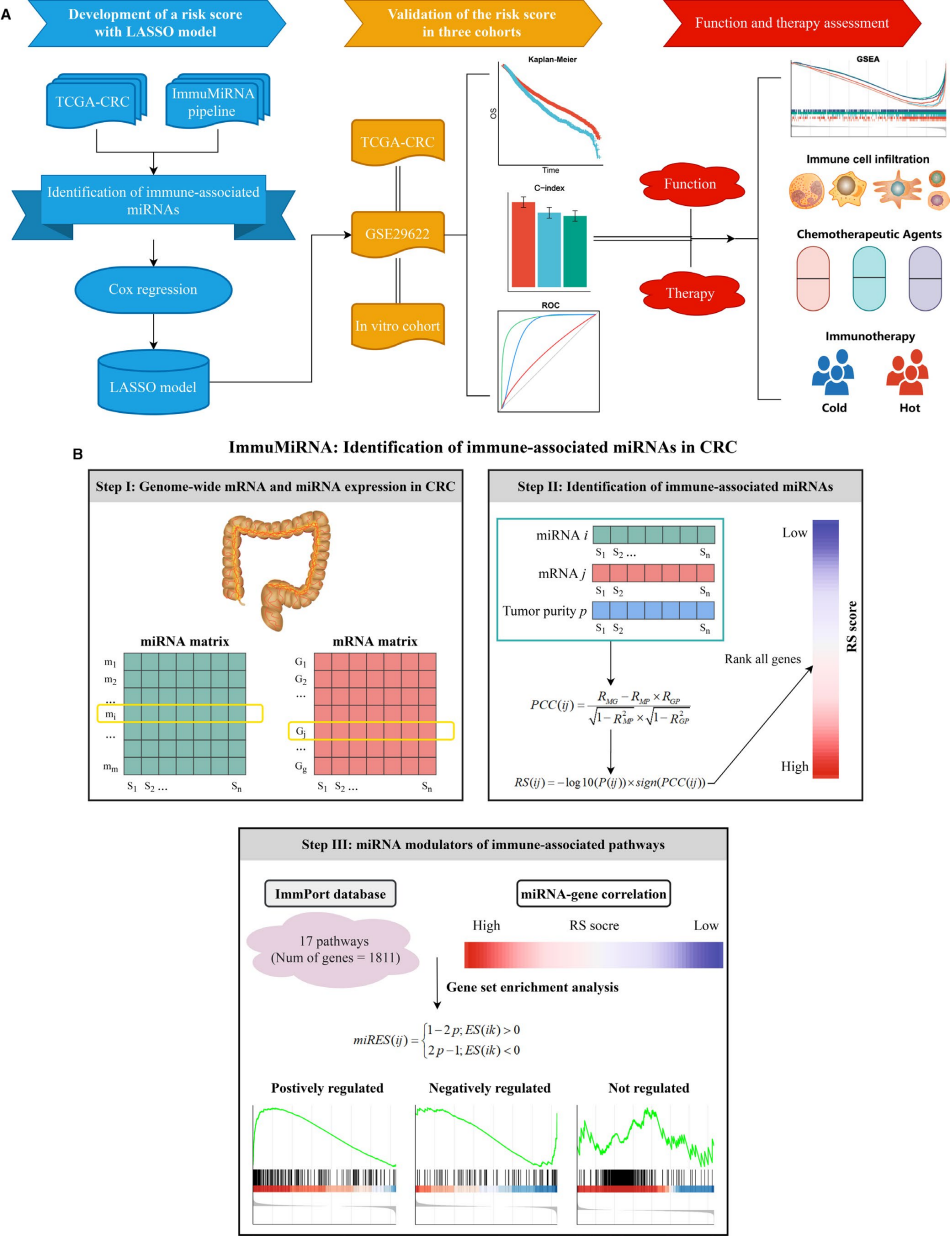
The overall study design and ImmuMiRNA pipeline. (A) Flow chart of our experimental design. (B) The ImmuMiRNA pipeline for identification of immune‐associated miRNAs in CRC

The overall workflow of our study is displayed in Figure 1A. The CRC data (n = 689) were enrolled from The Cancer Genome Atlas (TCGA) cohorts TCGA‐COAD (colon adenocarcinoma) and TCGA‐READ (rectum adenocarcinoma). ‘Level 3’ transcriptome profile (RNA‐Seq raw read count) and clinical information were retrieved from TCGA data portal (https://portal.gdc.cancer.gov/). Patients from TCGA were defined as TCGA‐CRC cohort. Human microRNA array GSE29622 including 65 CRC patients was extracted from Gene Expression Omnibus (GEO, http://www.ncbi.nlm.nih.gov/geo/). The normalized matrix file including miRNA expression profile and clinical information was directly downloaded. Patients were excluded if they 1) lacked mRNA or miRNA sequencing data; 2) did not have prognostic information; and 3) received neo‐adjuvant therapy. Detailed baseline data of CRC patients are displayed in Table S1.

### 2.2 Collection of immune‐associated genes

Human immune‐associated genes were retrieved from the ImmPort database (https://www.immport.org/). These gene sets were broadly used in immune‐associated studies.^12^, ^13^, ^14^ In aggregate, we summarized 1811 genes of 17 immune‐associated pathways for subsequent analyses.

### 2.3 Genome‐wide mRNA and miRNA expression in TCGA

The mRNA and miRNA raw read count from TCGA database was converted to transcripts per kilobase million (TPM) and reads per kilobase million (RPM), respectively. A further log2 transformation was performed because RNA‐seq data are often heavily right‐skewed in the linear scale. The GENCODE (https://www.gencodegenes.org/) and miRBase (http://www.mirbase.org/) were utilized to mRNA and miRNA annotations, respectively. The mRNAs and miRNAs with zero reads >50% of the samples were further excluded. In total, 16 985 mRNAs and 674 miRNAs were encompassed.

### 2.4 ImmuMiRNA: identification of immune‐associated miRNAs in CRC

To identify the latent miRNA modulators of immune‐associated pathways, we introduced an integrated algorithm that combines miRNA and gene expression data similar to ImmLnc.^15^ In short, all mRNAs were ranked by their correlation with a specific miRNA. The ranked gene list was further subjected to each immune‐associated pathway to explore whether the immune genes were enriched in the top or bottom of the list. The *miRES* score was calculated for each miRNA‐pathway pair. This process was repeated for all combinations of miRNAs and immune‐associated pathways. Based on a permutation test, all miRNA‐pathway pairs with significantly higher *miRES* scores were identified in CRC.

For each specific miRNA, we first ranked all mRNAs based on the correlation of their expression with this miRNA. The expression of miRNA *i* and gene *j* across *n* patients was labelled as M*(i)* = (*m_1_, m_2_, …, m_n_*) and *G(j)* = (*g_1_, g2, …, g_n_*), respectively. The tumour purity scores across *n* patients were labelled as *P* = (*p_1_, p_2_, …, p_n_*). We first calculated the partial correlation coefficient (PCC) between the expression of miRNA *i* and gene *j* by controlling the tumour purity as a covariable,

$$PCC\left(ij\right)=\frac{R_{\mathrm{MG}}‐R_{\mathrm{MP}}\times R_{\mathrm{GP}}}{\sqrt{1‐R_{\mathrm{MP}}^{2}}\times \sqrt{1‐R_{\mathrm{GP}}^{2}}}$$

where *R_MG_*, *R_MP_* and *R_GP_* are the correlation coefficients between the expression of miRNA *i* and protein‐coding gene *j*, the expression of miRNA *i* and tumour purity, and the expression of gene *j* and tumour purity, respectively. Moreover, we obtained the *P*‐value of the PCC, labelled as *P(ij)*, for each miRNA‐gene pair, and the rank score (*RS*) was calculated as follows:

RS(ij)=‐log10(P(ij))×sign(PCC(ij))

All genes were ranked based on *RS* indexes and further subjected to gene set enrichment analysis (GSEA). We mapped the genes of each immune‐associated pathway to the ranked gene list. For miRNA *i* and pathway *k*, we obtained the enrichment score (*ES*) and *P*‐value (adjusted by false discovery rate (FDR)) based on GSEA. Furthermore, following a previous study,^16^ the *P*‐value and the *ES* were combined to a *miRES* score, that is

$$miRES\left(ij\right)=\left\{\begin{array}{c} 1‐2p;ES(ik)>0\\ 2p‐1;ES(ik)<0 \end{array}\right.$$

where *ES(ik)* is the *ES* score between miRNA *i* and immune pathway *k*. Thus, the *miRES* score ranged from −1 to 1. We considered the miRNA‐pathway pairs with the absolute *miRES* >0.995 and FDR <0.05 as significant ones. To implement the pipeline described above, we developed a R package termed ‘ImmuMiRNA’ (https://github.com/Zaoqu‐Liu/ImmuMiRNA).

### 2.5 Construction and validation of the IAMIPS in public data sets

Before building the IAMIPS model, we transformed miRNA expression into z‐score in both TCGA‐CRC and GSE29622 data sets, which enhanced the comparability between different data sets. The TCGA‐CRC cohort served as the modelling set, and the GSE29622 served as the external validation set.

According to the immune‐associated miRNAs extracted above, we first performed univariate Cox regression analysis to select miRNAs that were significantly related to OS in TCGA‐CRC cohort. Given the aim to screen candidate miRNAs that were highly associated with OS and that the strictness of multiple testing correction might filter out some of these potential miRNAs, we included miRNAs with unadjusted *P*‐value <0.05 in the development of IAMIPS. A LASSO Cox regression approach was employed to determine candidates for the IAMIPS using ‘glmnet’ R package. The LASSO algorithm is a prevalent machine‐learning method, which is widely applied to the Cox proportional hazard regression models for prognostic analysis.^17^, ^18^ To determine the optimal values of lambda, we used 10‐fold cross‐validations with the 1‐standard error (SE) criteria,^18^ and the optimal lambda is the largest value for which the partial likelihood deviance is within one SE of the smallest value of partial likelihood deviance. Subsequently, based on this lambda value, the miRNAs with non‐zero coefficients were selected to construct the prediction model. The risk score for each patient was calculated with the LASSO model weighting coefficient as follows:

$$Risk\;score=\sum_{i=1}^{n}Exp_{i}\times Coef_{i}$$

where *n* is the number of key miRNAs, *Exp_i_* is the expression of miRNA *i*, and *Coef_i_* is the LASSO coefficient of miRNA *i*. The optimal risk score cut‐off value was determined by ‘survminer’ R package in the TCGA‐CRC cohort. Using this cut‐off value, the patients were divided into high‐risk and low‐risk groups. Human microRNA array GSE29622 served as external validation.

### 2.6 Human CRC specimens

This study was approved by the First Affiliated Hospital of Zhengzhou University. A total of 40 paired CRC tissues and matched adjacent non‐tumour tissues were obtained from patients after receiving surgical resection at The First Affiliated Hospital of Zhengzhou University. None of the patients received any preoperative chemotherapy or radiotherapy. Written informed consent was obtained from all patients. The inclusion criteria were as follows: no preoperative chemotherapy, radiotherapy or targeted therapy; no other types of tumours; and no autoimmune diseases. The specimens obtained during surgery were immediately snap‐frozen in liquid nitrogen and stored at −80℃ until RNA extraction. Clinical staging of the specimens was based on NCCN (2019) guidelines. Detailed baseline data of CRC patients are displayed in Table S1.

### 2.7 Validated the IAMIPS in vitro experiment

Total RNA was isolated from CRC tissues and paired adjacent non‐tumour tissues with RNAiso Plus reagent (Takara, Dalian, China) according to the manufacturer's instructions. RNA quality was evaluated using a NanoDrop One C, and RNA integrity was assessed using agarose gel electrophoresis. An aliquot of 1 µg of total RNA was reverse‐transcribed into complementary DNA (cDNA) according to the manufacturer's protocol using the miRNA reverse transcription Kit (TaKaRa BIO). Quantitative real‐time PCR (qRT‐PCR) was performed using SYBR Assay I Low ROX (Eurogentec) and SYBR® Green PCR Master Mix (Yeasen) to detect the expression. The data were normalized to the expression of U6. The sequences of the primers were as follows: *miR‐194‐3p*, forward 5’‐ACACTCCCAGUGGGGCUG‐3’ and reverse 5’‐CAGAUAACAGTTGAGAGTACAT‐3’; *miR‐216a‐5p*, forward 5’‐GGGTAATCTCAGCTGGCAA‐3’ and reverse 5’‐CAGTGCGTGTCGTGGAGT‐3’; *miR‐3677‐3p*, forward 5’‐CAGTGGCCAGAGCCCTGCA‐3’ and reverse 5’‐GAACATGTCTGCGTATCTC‐3’; and *U6*, forward 5’‐CTCGCTTCGGCAGCACA‐3’ and reverse 5’‐AACGCTTCACGAATTTGCGT‐3’. Based on the miRNA expression from qRT‐PCR, we further validated the IAMIPS in our CRC cohort.

### 2.8 Gene set enrichment analysis

To explore the potential molecular mechanisms underlying the IAMIPS, GSEA was performed to identify enriched terms related to Kyoto Encyclopedia of Genes and Genomes (KEGG) pathway and biological process of gene ontology (GO) between high‐risk and low‐risk groups. Gene set permutations were performed 1000 times for each analysis. Gene sets with FDR <0.01 were considered to be significantly enriched.

Single sample gene set enrichment analysis (ssGSEA) was applied to quantify the relative abundance of 28 immune cells in the tumour microenvironment of CRC. The gene set for marking each cell was obtained from the research of Charoentong, which stored various human immune cell subtypes including activated CD8+ T cell, activated dendritic cell, natural killer T cell, and macrophage (Table S2).^19^

### 2.9 Prediction the clinical chemotherapeutic response

To assess the drug response in TCGA‐CRC cohort, we downloaded the imputed tumour response to 138 anticancer drugs in CRC patients from a previous study.^20^ Drug sensitivity was quantified by half‐maximal inhibitory concentration (IC50); the lower the IC50, the more sensitive the drug. We identified tumour drugs with specific sensitivity in high‐risk and low‐risk groups using the following criteria: 1) a Pearson correlation was calculated for each drug's IC50 and risk score. Drugs with absolute correlation coefficient >0.3 and FDR <0.05 were retained; 2) t test was performed to compare the sensitivity difference between high‐risk and low‐risk groups. Drugs with absolute log2 fold change value >0.5 and FDR <0.05 were included; and 3) IAMIPS‐related drugs were determined by the intersection of the above two results.

### 2.10 Evaluation of the immunotherapy response

The Tumor Immune Dysfunction and Exclusion (TIDE) algorithm was employed to predict the immunotherapy response of each patient.^21^ TIDE algorithm was a computational method to model two primary mechanisms of tumour immune evasion: the induction of T cell dysfunction in tumours with high infiltration of cytotoxic T lymphocytes (CTL) and the prevention of T cell infiltration in tumours with low CTL level. Next, the Subclass Mapping (SubMap) method was utilized to evaluate the similarity between the risk groups and the patients on immunotherapy.^22^ The SubMap employs GSEA algorithm to deduce the extent of commonality of the two groups. Adjusted *P*‐values were used to assess the similarity, and the lower adjusted *P*‐values suggested the higher similarity.

### 2.11 Statistical analysis

Independent sample t test and paired t test were utilized to compare the miRNA expression difference in public data sets and 40 paired tissues, respectively. The Kaplan‐Meier method and the log‐rank test were used to estimate the different OS between high‐risk and low‐risk groups. Univariate Cox regression analysis was used to calculate the hazard ratios (HRs). The receiver operating characteristic (ROC) curves were plotted by ‘timeROC’ R package. Area under the ROC curve (AUC) and Harrell's concordance index (C‐index) were employed to evaluate the performance of the IAMIPS in predicting OS. ROC curves of different indicators were compared using the *compare()* function in ‘timeROC’ R package. The optimal cut‐off value of risk score was determined by ‘survminer’ R package. All *P*‐values were two‐sided, with *P* < 0.05 as statistically significant. Adjusted *P‐value* was obtained by Benjamini‐Hochberg (BH) multiple test correction. All data processing, statistical analysis and plotting were conducted in R 4.0.2 software.

## 3 RESULTS

### 3.1 Identification of immune‐associated miRNAs in CRC

To identify miRNAs that were related to immune‐associated pathways, we developed a three‐step integrated algorithm framework termed ImmuMiRNA (Figure 1B). ImmuMiRNA systematically deduces candidate miRNA regulators of immune‐associated pathway activity from miRNA and gene expression profiles. One hypothesis is that if a specific miRNA plays critical roles in immune regulation, then its related genes should be enriched in the top or bottom of immune‐associated pathways. In short, ImmuMiRNA identifies the miRNA regulators by three steps (Figure 1B). First, we extracted the miRNA and mRNA expression profiles of the same CRC patients. Second, the tumour purity of each sample was evaluated and all genes were ranked according to the rank score (RS) for each candidate miRNA. Third, we calculated the enrichment score of each miRNA in the immune pathway (*miRES*) based on GSEA. The *P*‐value of GSEA was transformed to a *miRES* score and the miRNA‐pathway pairs with *miRES* >0.995 and FDR <0.05 were screened (Figure 1B).

**FIGURE 2:**
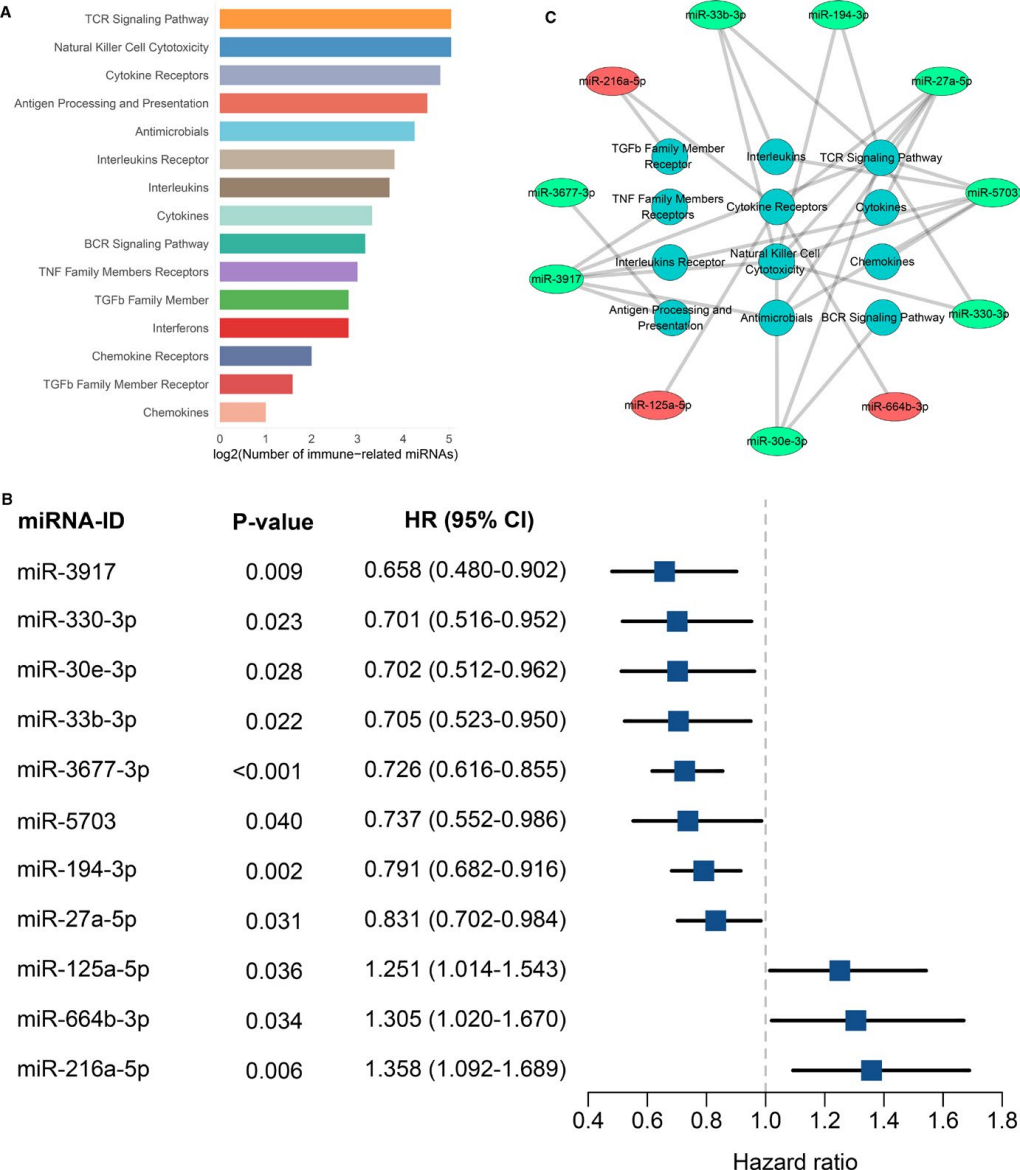
Identification of immune‐associated miRNAs in TCGA‐CRC cohort. (A) The number of miRNAs significantly associated with immune‐related pathways. (B) Univariate Cox regression revealed 11 miRNAs with significant prognostic significance. (C) Various immune‐related pathways that these 11 prognostically relevant miRNAs may be involved in

By virtue of the ImmuMiRNA pipeline, we identified a total of 97 immune‐associated miRNAs, which accounted for 10% of all miRNAs in the TCGA‐CRC cohort (Table S2). A higher number of miRNAs were correlated with the ‘T cell receptor signalling’, ‘natural killer cell cytotoxicity’, ‘cytokine receptors’ and ‘antigen processing and presentation’ pathways (Figure 2A). Currently, restoring or enhancing the activity of T cells and natural killer cells is considered to be the mainstay of immunotherapy.^23^ These miRNA regulators will be a resource for dissecting the immune regulation in CRC. Univariate Cox regression analysis between each of the 97 miRNAs and OS is shown in Table S3. A total of 11 miRNAs significantly correlated with OS were identified, of which 8 were protective factors and 3 were risk factors (*P* < 0.05; Figure 2B). These OS‐associated miRNAs demonstrated correlations with a variety of immune‐associated pathways, which suggested that activation and inhibition of various immune pathways were significantly correlated with OS in patients (Figure 2C).

### 3.2 Construction and evaluation of the IAMIPS

**FIGURE 3:**
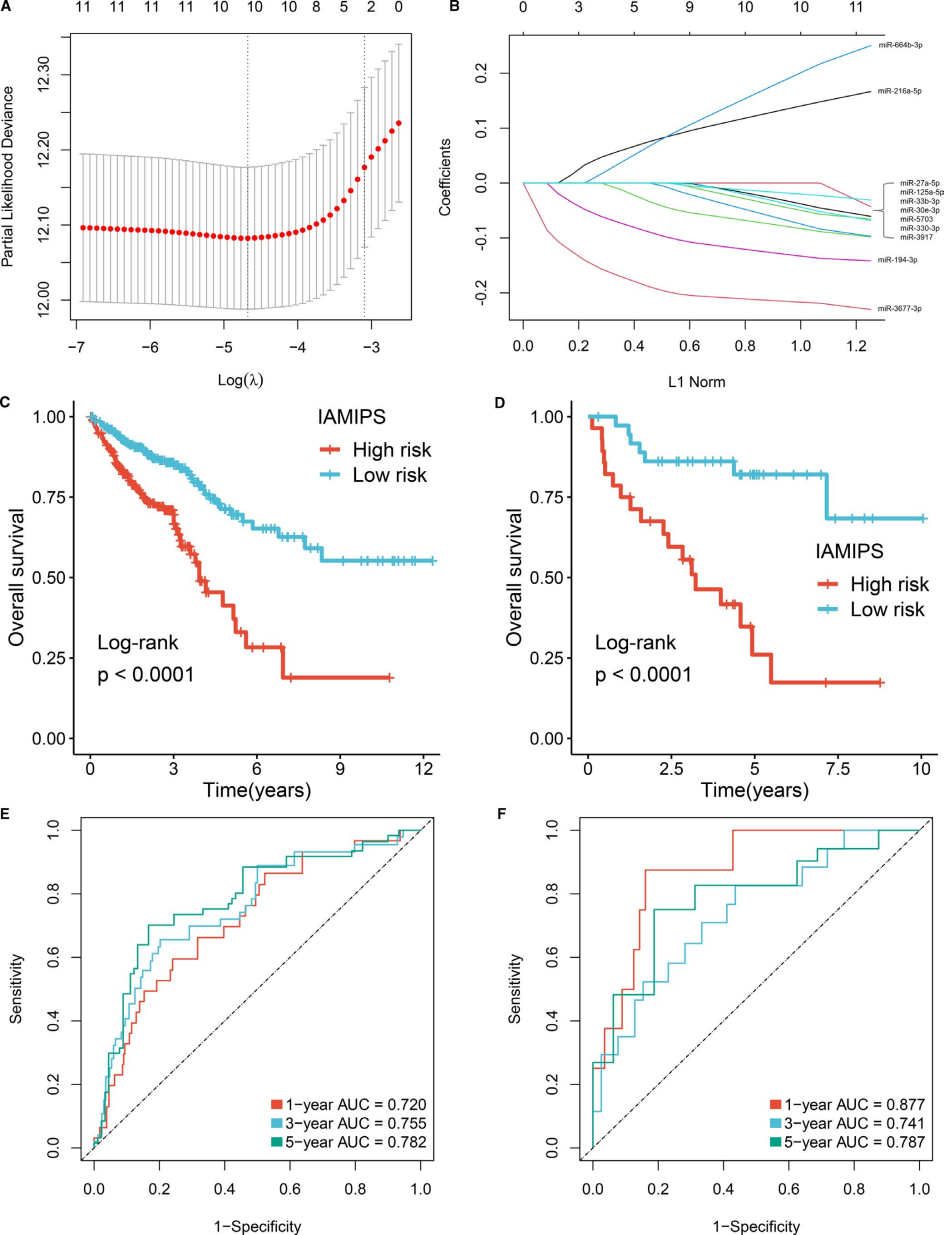
Construction and evaluation of the IAMIPS. (A) Ten‐time cross‐validations to tune the parameter selection in the LASSO model. The two dotted vertical lines are drawn at the optimal values by minimum criteria (left) and 1−SE criteria (right). (B) LASSO coefficient profiles of the candidate miRNAs for IAMIPS construction. (C‐D) Kaplan‐Meier curves for OS according to the IAMIPS in TCGA‐CRC (C) and GSE29622 (D) cohorts. (E‐F) Time‐dependent ROC analysis of the IAMIPS for 1‐, 3‐ and 5‐year OS in TCGA‐CRC (E) and GSE29622 (F) cohorts

The 11 OS‐associated miRNAs were selected to construct an IAMIPS. We employed a LASSO Cox regression model and identified three miRNAs that were strongly predictive of OS, including *miR‐216a‐5p*, *miR‐194‐3p* and *miR‐3677‐3p* (Figure 3A,B). The three miRNAs also demonstrated significant differences between tumours and normal tissues (*P* < 0.05; Figure S1A). Next, a risk score for IAMIPS was calculated using a formula that including the three miRNAs weighted by their regression coefficients in a penalized Cox model as follows: Risk score =0.015 × the expression of *miR‐216a‐5p* ‐ 0.035 × the expression of *miR‐194‐3p* ‐ 0.124 × the expression of *miR‐3677‐3p*.

Using the optimal cut‐off value (0.05) for the IAMIPS, we divided 635 patients in the TCGA‐CRC cohort and 65 patients in the GSE29622 into high‐risk and low‐risk groups, respectively. Patients in the high‐risk group had a shorter OS than the low‐risk group (log‐rank test, both *P* < 0.05; Figure 3C,D). We questioned the generality of IAMIPS for various clinicopathological characteristics in CRC; thus, the stratified survival analysis was further performed. After stratification for age, gender, clinical stage and microsatellite instability, the signature remained a powerful independent predictor for OS and was suitable to the vast majority of CRC patients (log‐rank test, *P* < 0.05; Figure S1B).

The time‐dependent ROC and C‐index were applied to evaluate the performance of the signature. The AUCs of 1, 3 and 5 years were 0.720, 0.755 and 0.782 in the TCGA‐CRC cohort; and 0.877, 0.741 and 0.787 in the GSE29622 cohort (Figure 3E,F). The C‐index was 0.725 [95% CI: 0.700 ~ 0.754] and 0.747 [95% CI: 0.643 ~ 0.851] in the TCGA‐CRC cohort and the GSE29622 cohort, respectively. To further evaluate the predictive performance of the IAMIPS for OS, we first determined whether the IAMIPS outperformed each miRNA. The ROC results demonstrated the IAMIPS displayed better performance than each miRNA at predicting 1‐, 3‐ and 5‐year OS in two cohorts (*P* < 0.05; Figure S2A,B). Traditional clinical stage is currently the main method to assess the prognosis of CRC patients. Therefore, we next evaluated the prognostic performance of AJCC stage versus that of the IAMIPS. The IAMIPS also displayed better accuracy than traditional AJCC stage in two cohorts (both *P* < 0.05; Figure S2A,B).

### 3.3 Validation of the IAMIPS in our cohort

**FIGURE 4:**
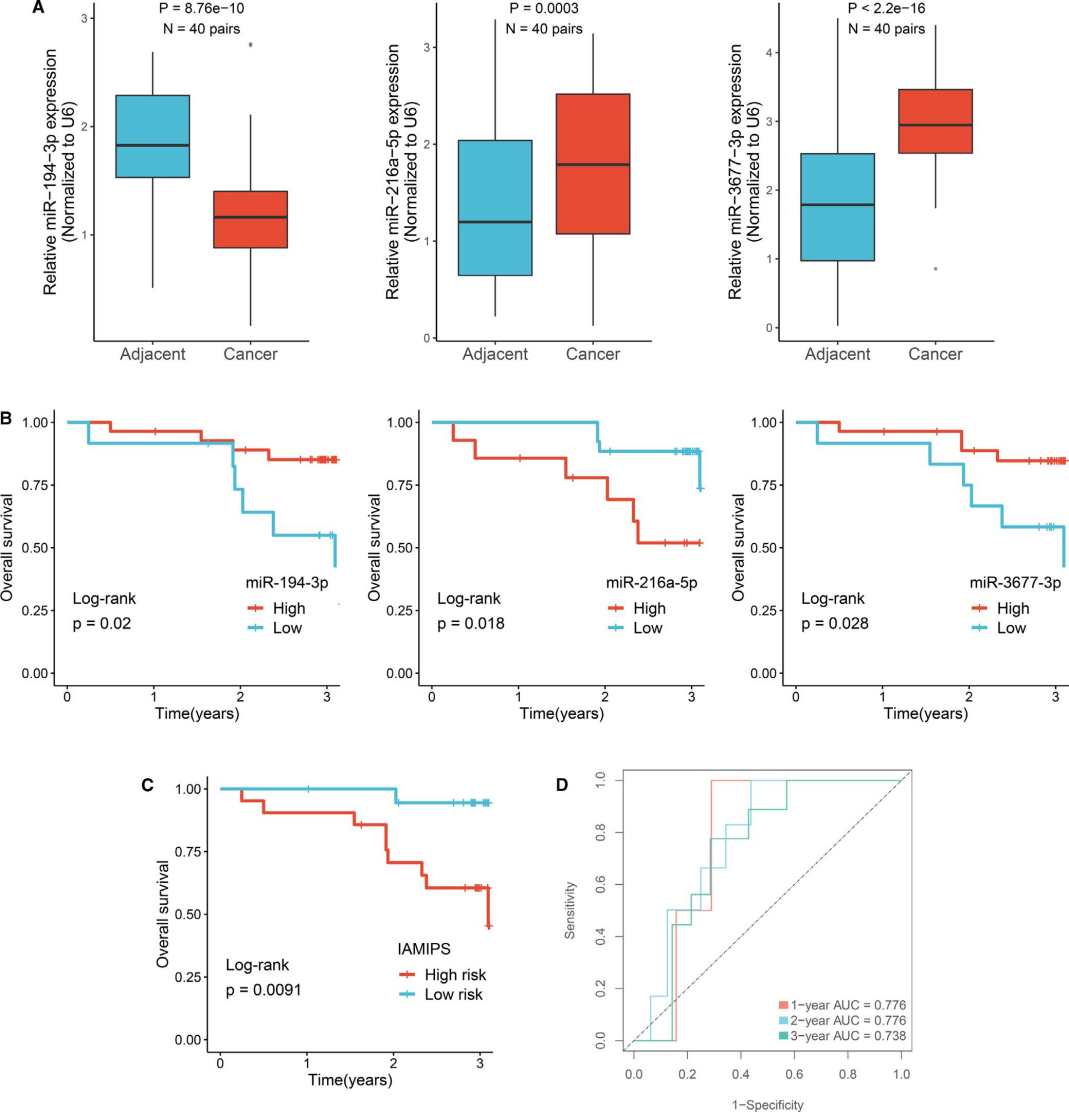
Validation of the IAMIPS in our cohort. (A) qRT‐PCR displayed the expression level of miR‐194‐3P, miR‐216a‐5p and miR‐3677‐3p between CRC tissues and their corresponding adjacent non‐tumour tissues. (B) Kaplan‐Meier curves for OS according to the expression of miR‐194‐3P, miR‐216a‐5p and miR‐3677‐3p. (C) Kaplan‐Meier curves for OS according to the IAMIPS. (D) Time‐dependent ROC analysis of the IAMIPS for 1‐, 2‐ and 3‐year OS

We enrolled 40 CRC tissues and 40 paired non‐tumour tissues from the First Affiliated Hospital of Zhengzhou University. Follow‐up was concluded three years after surgery. Table S1 shows their clinical characteristics. qRT‐PCR assay was performed in 40 pairs of CRC tissues and matched adjacent non‐tumour tissues. In line with above results, the three miRNAs displayed significantly expression difference in tumour relative to normal tissues (*P* < 0.05; Figure 4A). Patients with low expression *miR‐194‐3p* and *miR‐3677‐3p* or high expression of *miR‐216a‐5p* tended to indicate an adverse OS (log‐rank test, *P* < 0.05; Figure 4B).

We determined another optimal cut‐off value (0.008) for qRT‐PCR assay and further divided 40 patients into high‐risk and low‐risk groups. Kaplan‐Meier survival analysis demonstrated patients in the high‐risk group showed worse OS than the low‐risk group (log‐rank test, *P* < 0.05; Figure 4C). The AUCs of 1, 2 and 3 years was 0.776, 0.776 and 0.738, respectively (Figure 4D). The C‐index was 0.759 [95% CI: 0.679 ~ 0.839]. These results suggested the IAMIPS model possessed a robust and reliable predictive performance for OS.

### 3.4 Inflammatory profiles and immune checkpoint landscape of IAMIPS

**FIGURE 5:**
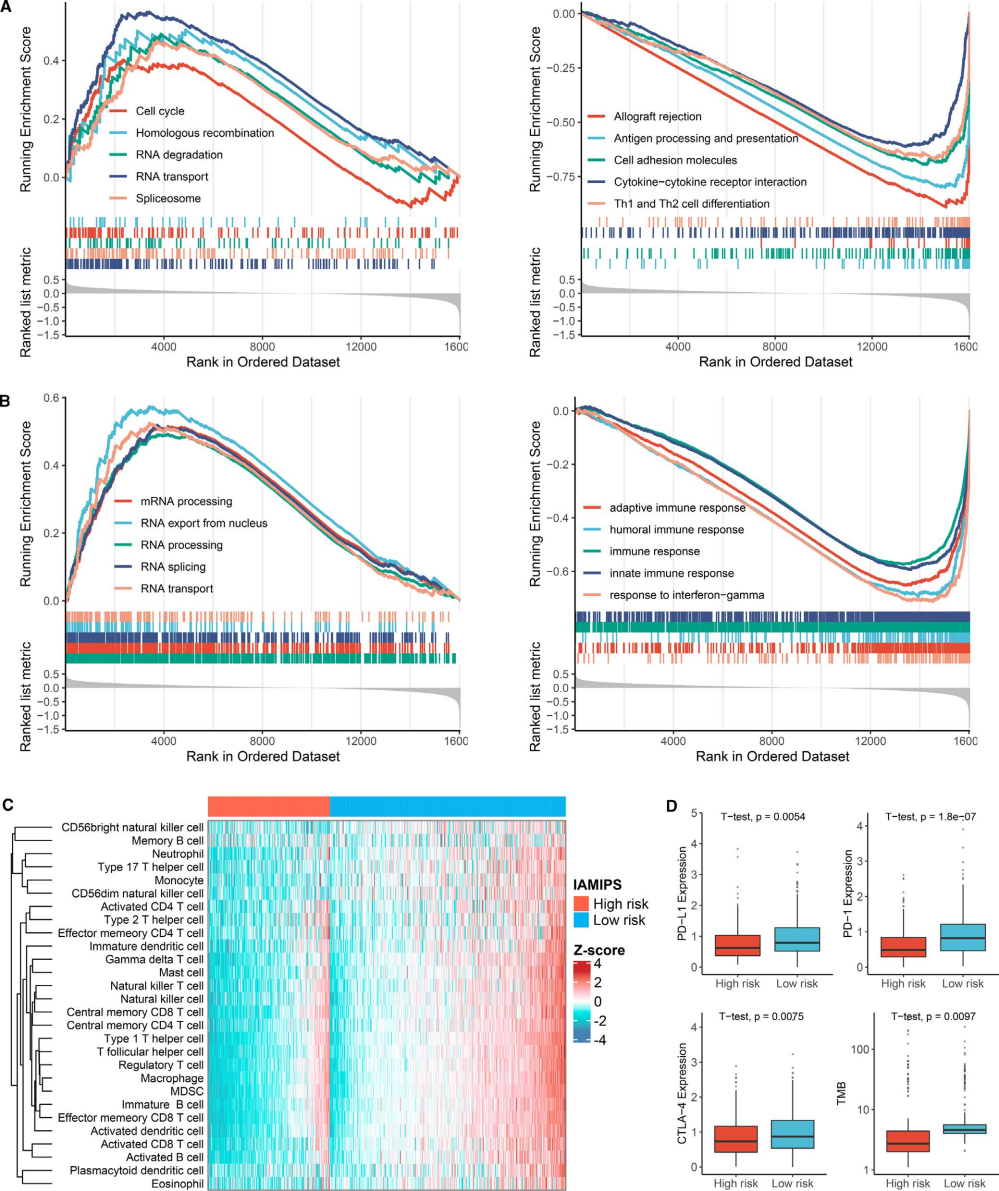
Functional and immune cell infiltration assessment. (A) GSEA results demonstrated the KEGG pathways enriched in the high‐risk and low‐risk groups, respectively. (B) GSEA results demonstrated the GO terms enriched in the high‐risk and low‐risk groups, respectively. (C) Heatmap of 28 immune cell infiltration abundance in two groups. (D) The distribution difference of PD‐L1, PD‐1, CTLA‐4 expression and TMB between the high‐risk and low‐risk groups

GSEA was performed to better understand the potential molecular mechanisms underlying the IAMIPS. As shown in Figure 5A,B, the high‐risk group was mainly associated with tumour proliferation such as cell cycle, spliceosome and mRNA processing. The low‐risk group was mainly associated with immunology such as antigen processing and presentation, cytokine‐cytokine receptor interaction and adaptive immune response. These results explained the worse prognosis of patients in the high‐risk group. Intriguingly, a large number of immune‐associated pathways were enriched in the low‐risk group, which indicated patients in the low‐risk group had favourable immune infiltration status. Therefore, we further adopted the ssGSEA algorithm to assess the relative infiltration abundance of 28 immune cell types. Consistent with the above results, the abundance of immune cell infiltration in the low‐risk group was significantly higher than the high‐risk group, such as B cells, activated CD4+/CD8+ T cells, dendritic cells and natural killer cells (*P* < 0.05; Figure 5C, Figure S3). Overall, the high‐risk group was significantly correlated with tumour proliferation and presented inferior immune cell infiltration, suggesting an ‘immune‐cold’ phenotype, while the low‐risk group enriched plenty of immune‐associated pathways and displayed abundant immune cell infiltration, suggesting an ‘immune‐hot’ phenotype. These findings have latent implications for the rational design of combination immunotherapy strategies. For patients in the low‐risk group, ICIs might be applied to enhance the pre‐existing antitumour immunity of these patients and further prolong their survival. Conversely, for patients in the high‐risk group, the response of ICIs alone might be unsatisfied due to unfavourable immune activation. In addition, we compared the differences in popular indicators of immunotherapy such as *PD‐1*, *PD‐L1*, *CTLA‐4* and TMB between the two groups. As expected, the low‐risk groups all displayed higher expression level of these indicators compared with the high‐risk groups (*P* < 0.05; Figure 5D), which suggested that patients in the low‐risk group was more likely to benefit from available immunotherapeutic drugs such as atezolizumab, pembrolizumab and ipilimumab.^5^

### 3.5 Implications of IAMIPS on CRC chemotherapy

**FIGURE 6:**
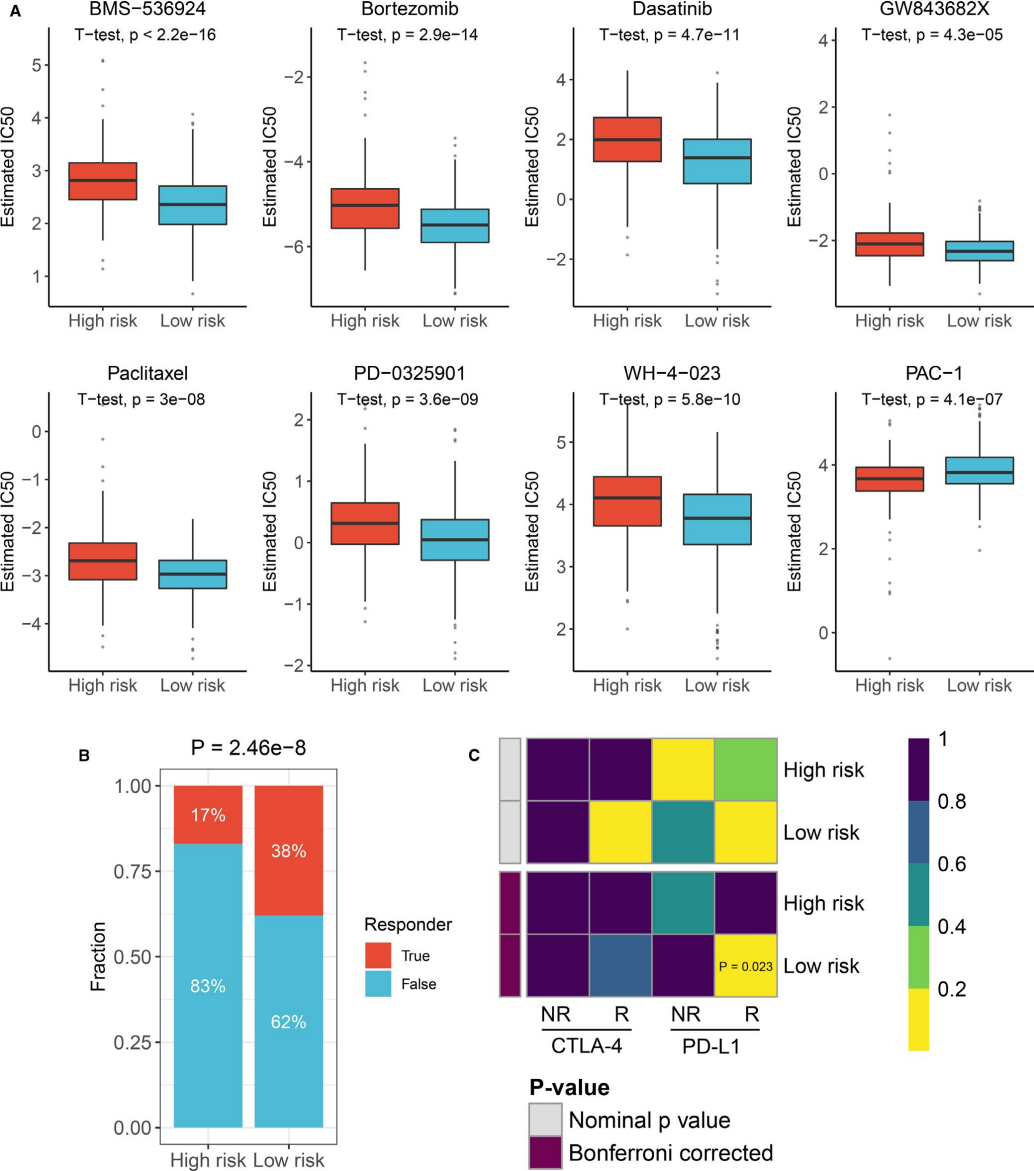
Implications of IAMIPS on CRC treatment. (A) The estimated IC50 level of IAMIPS‐related antineoplastic drugs between the high‐risk and low‐risk groups. (B) Distribution of the immunotherapy response results predicted by TIDE algorithm between the high‐risk and low‐risk groups. (C) SubMap analysis manifested that the low‐risk group could be more sensitive to the anti‐PD‐1 therapy (Bonferroni‐corrected *P* =.023)

We further identify several IAMIPS‐related antineoplastic drugs. As shown in Figure 6A, we observed that patients in the low‐risk group were more sensitive to BMS‐536924, bortezomib, dasatinib, GW843682X, paclitaxel, PD‐0325901 and WH‐4‐023 and patients in the high‐risk group were more sensitive to PAC‐1 (*P* < 0.05). These drugs provided a resource for precision chemotherapy in two groups.

Since we identified ‘immune‐hot’ and ‘immune‐cold’ phenotypes in two groups, further immunotherapy evaluations were performed. Using the TIDE tool at Harvard University, we observed patients in the low‐risk group had more immunotherapy response rate than the high‐risk group (38% vs. 17%; *P* < 0.05; Figure 6B). In addition, SubMap analysis indicated the low‐risk group displayed high similarity with patients who responded to anti‐PD‐1 therapy (*P* < 0.05; Figure 6C). These results further proved patients in the low‐risk group could benefit more from immunotherapy, particularly anti‐PD‐1 therapy.

## 4 DISCUSSION

Accumulating evidence suggests that miRNAs are critical for immune regulation. Nevertheless, only a few examples have been identified so far. In the present study, we reported the use of the ImmuMiRNA algorithm to systematically identify the miRNA regulators that latently regulate immune‐associated pathways. Based on three immune‐associated miRNAs that strongly predicted OS, we developed and externally validated a novel prognostic tool that improved the ability to predict OS of patients with CRC. Our results displayed that IAMIPS could successfully divide patients into high‐risk and low‐risk groups with significant differences in OS. The IAMIPS was proved to be an independent prognostic factor as well as performed better than single miRNA and clinical stage. Furthermore, patients in the high‐risk group were dramatically correlated with tumour proliferation and presented inferior immune cell infiltration, suggesting an ‘immune‐cold’ phenotype, while patients in the low‐risk group enriched plenty of immune‐associated pathways and displayed abundant immune cell infiltration, suggesting an ‘immune‐hot’ phenotype. These results indicated that patients in the low‐risk group might benefit more from immunotherapy.

MiRNAs are emerging as critical regulators of gene expression in the immune system and play essential roles in the development and progression of CRC.^24^ Exploring the miRNAs in immunomodulatory network as well as their translational value is necessary for understanding the molecular mechanisms of CRC carcinogenesis and improving the clinical management of CRC. In this study, a three‐step framework termed ImmuMiRNA was proposed for systematically deducing candidate miRNA regulators of immune‐associated pathways. An R package was further developed, ImmuMiRNA (https://github.com/Zaoqu‐Liu/ImmuMiRNA), to nimbly implement the algorithm pipeline. A total of 97 immune‐associated miRNAs were identified, and a higher number of miRNAs were correlated with ‘T cell receptor signalling’, ‘natural killer cytotoxicity’, ‘cytokine receptor’ and ‘antigen processing and presentation’ pathways. Restoring or enhancing the activity of T cells and natural killer cells is currently the mainstay of immunotherapy,^23^ and these miRNA regulators will be a resource for dissecting the immune regulation in CRC. Immune‐associated miRNAs also displayed significant impacts on the prognosis of patients with CRC. In our study, eleven miRNAs that dramatically related to OS were further identified.

An ideal machine‐learning model should have fewer variables and achieve better efficacy.^25^ Hence, we applied the LASSO algorithm, which was known to select key variables to avoid overfit of model.^17^, ^18^ Ultimately, three key miRNAs including *miR‐216a‐5p*, *miR‐194‐3p* and *miR‐3677‐3p* were determined. In vitro experiments further demonstrated their abnormal expression and prognosis significance in CRC. Based on these three miRNAs, a simple model, IAMIPS, was further developed and validated in TCGA‐CRC and GSE29622 cohorts. The IAMIPS performed better than single miRNAs and traditional clinical stage. The excellent performance in evaluating OS of patients with CRC in two independent cohorts demonstrated IAMIPS was robust biomarker. Moreover, to enhance the clinical transformation of IAMIPS, we used qRT‐PCR method to quantify the three miRNAs in 40 CRC samples from our hospital. Consequently, the results were consistent and showed good performance, which suggested IAMIPS was a promising and convenient tool for evaluating OS of patients with CRC. In clinical practice, qRT‐PCR quantification of only three miRNAs in CRC tissues can assess the prognostic risk of patients.

In addition, we found the high‐risk group was significantly correlated with tumour proliferation and presented inferior immune cell infiltration, suggesting an ‘immune‐cold’ phenotype, while the low‐risk group enriched plenty of immune‐associated pathways and displayed abundant immune cell infiltration, suggesting an ‘immune‐hot’ phenotype. The above suggested that IAMIPS can well stratify CRC patients based on their immune status, which had implications for immunotherapy in CRC. Solid tumours in ‘immune‐hot’ status tend to have better immunotherapy response.^26^ Hence, patients in the low‐risk group might benefit more from immunotherapy. Bioinformatics algorithms including TIDE and SubMap methods further validated this conclusion. However, the limitation of our study is evaluating the immunotherapy response using bioinformatics algorithms rather than conducting large‐scale immunotherapy clinical trials. In spite of this, the above results were highly consistent in terms of functional analysis and predictive results, which indicates that our results are relatively reliable. Moreover, we identified latent antitumour drugs significantly associated with IAMIPS, hoping to provide additional reference for antitumour therapies of patients with different IAMIPS risk.

In summary, we proposed a novel algorithm, ImmuMiRNA, which can systematically identify the miRNA regulators that latently regulate immune‐associated pathways in CRC. The IAMIPS will facilitate the complex underlying mechanisms between immune‐associated miRNAs and prognosis of CRC and may advance optimize immunotherapies for patients with CRC.

## CONFLICT OF INTEREST

The authors declare that they have no conflicts of interest.

## AUTHOR CONTRIBUTION

**Zaoqu Liu:** Conceptualization (equal); Investigation (equal); Methodology (equal); Writing‐original draft (equal); Writing‐review & editing (equal). **Taoyuan Lu:** Investigation (equal); Methodology (equal); Writing‐original draft (equal). **Yanli Wang:** Writing‐original draft (equal); Writing‐review & editing (equal). **Dechao Jiao:** Data curation (equal); Investigation (equal). **Zhao‐nan Li:** Writing‐original draft (equal). **Libo Wang:** Writing‐original draft (equal). **Long Liu:** Writing‐original draft (equal). **Chunguang Guo:** Writing‐original draft (equal). **Yanan Zhao:** Investigation (equal); Methodology (equal). **Xinwei Han:** Conceptualization (equal); Writing‐review & editing (equal).

## ETHICS APPROVAL AND CONSENT TO PARTICIPATE

The human cancer tissues used in this study were approved by Ethnics Committee of The First Affiliated Hospital of Zhengzhou University in 19 December 2019, and the TRN is 2019‐KW‐423.

## Supporting information

Fig S1

Fig S2

Fig S3

Table S1‐S3

## Data Availability

The data used to support the findings of this study are available from the corresponding author upon request.
