# Supplementary material for: Establishment and experimental validation of an immune miRNA signature for assessing prognosis and immune landscape of patients with colorectal cancer
Source: J Cell Mol Med. 2021 Jun 7;25(14):6874–86. doi: 10.1111/jcmm.16696 (PMC8278100; doi:10.1111/jcmm.16696)
Supplement: Supplementary file 1 — Fig S1 [file JCMM-25-6874-s001.docx]

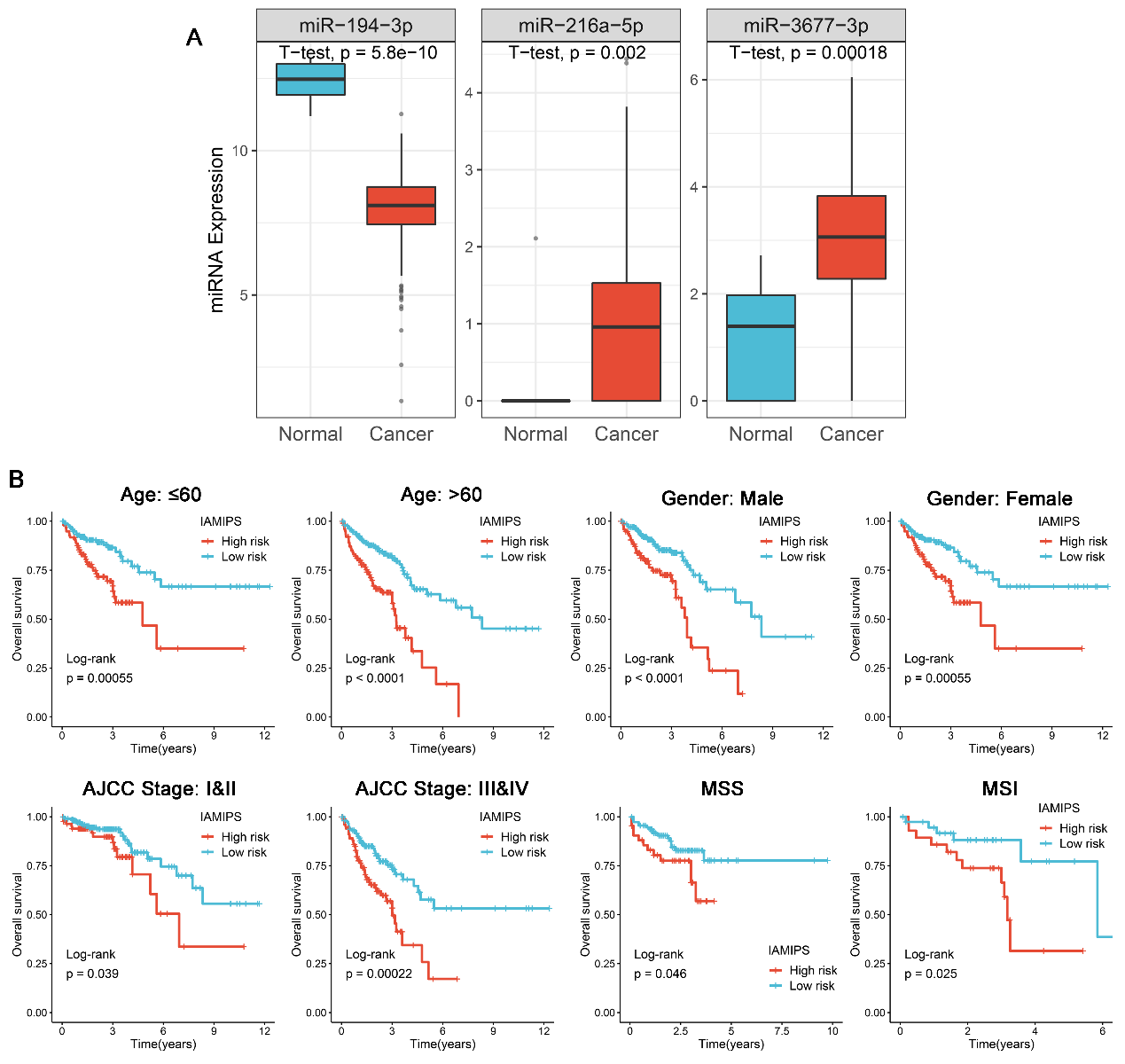


**Figure S1**. The expression level of miRNA and the stratified survival analysis of IAMIPS. **A**. The expression level of miR-194-3p, miR-216a-5p, and miR-3677-3p between CRC and normal tissues. **B**. The stratified survival analysis of IAMIPS for age, gender, clinical stage, and microsatellite instability.
