# Supplementary material for: Establishment and experimental validation of an immune miRNA signature for assessing prognosis and immune landscape of patients with colorectal cancer
Source: J Cell Mol Med. 2021 Jun 7;25(14):6874–86. doi: 10.1111/jcmm.16696 (PMC8278100; doi:10.1111/jcmm.16696)
Supplement: Supplementary file 2 — Fig S2 [file JCMM-25-6874-s003.docx]

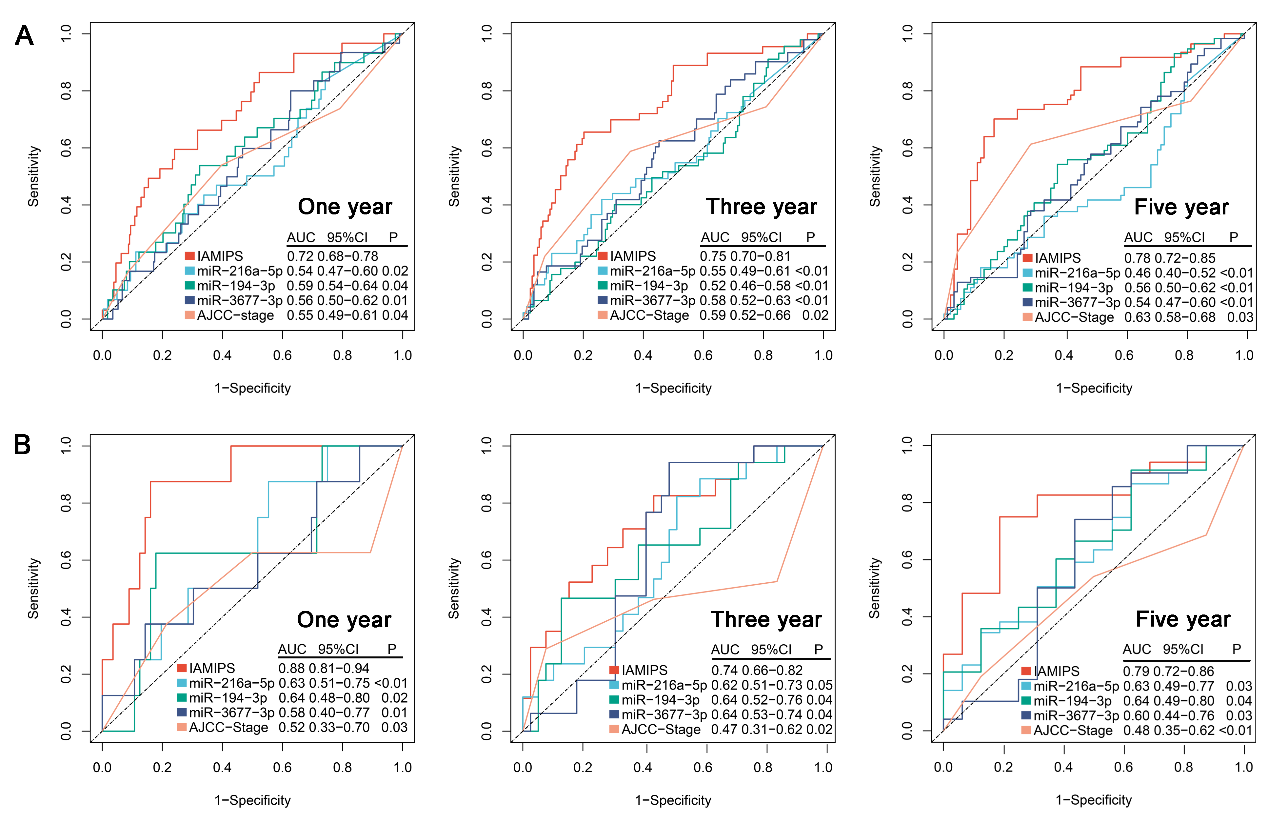


**Figure S2**. Time-dependent ROC analysis compared the IAMIPS signature, individual miRNAs, and AJCC stage. **A-B**. Time-dependent ROC analysis compared the IAMIPS signature, individual miRNAs, and AJCC stage for 1-, 3-, and 5-year OS in TCGA-CRC (**A**) and GSE29622 (**B**) cohorts.
