# Supplementary material for: Establishment and experimental validation of an immune miRNA signature for assessing prognosis and immune landscape of patients with colorectal cancer
Source: J Cell Mol Med. 2021 Jun 7;25(14):6874–86. doi: 10.1111/jcmm.16696 (PMC8278100; doi:10.1111/jcmm.16696)
Supplement: Supplementary file 3 — Fig S3 [file JCMM-25-6874-s004.docx]

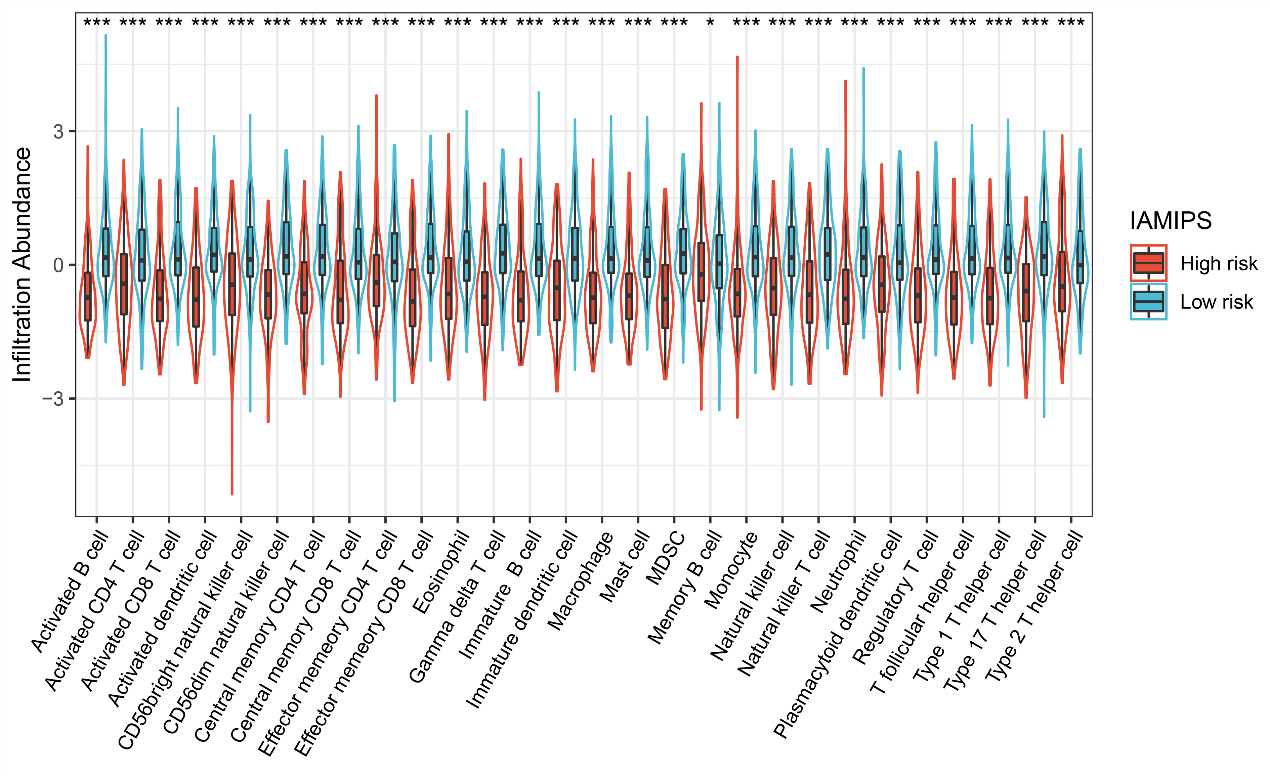


**Figure S3**. Boxplot for comparing the infiltration difference of 28 immune cells between the high-risk and low-risk groups.
